# Health Tract, No. 298

**Published:** 1867-08

**Authors:** 


HEALTH TRACT, No. 298.

### VENTILATING CELLARS.

The air of cellars is very generally heavy and damp, causing whatever is in them to be soon covered with mould, besides sending up poisonous gases through the chambers above, and whatever plan will serve even in part to purify the air of the cellar by conveying its noisome exhalations without the building and thus making room for a dryer and purer air from without, will contribute greatly to the comfort and enjoyment and health fulness of any household, and that plan is best which is self-acting and does not depend upon the attention of servants or bad housekeepers. Let a common stove-pipe with one elbow be arranged with one open end three or four inches above the cellar floor, passing through the ceiling of the cellar and floor of the kitchen or other apartment where there is a constant fire, and be introduced into the flue within a foot of the kitchen ceiling by the elbow; at some point within reach of this stove-pipe a damper should be attached which, when desired, would stop the draft from the cellar.

A correspondent of the Scientific American suggests a modification of this arrangement, which is given in his own words: "In my sitting room immediately over the cellar, I have a small, cast iron, air-tight, wood burning stove, with three and a half feet of six inch pipe connected through a thimble with the chimney flue at about one foot from the stove. I have a T connection with the stove pipe, with pipe of the same size, passing through the floor and reaching to within a foot of the cellar floor. At the top of this pipe, close to the connection with the stove pipe, there is a valve which regulates the draft of cold air taken from the cellar. The opening in the floor is half an inch larger than the pipe. The vacuum produced in the cellar by the draft in the chimney flue thus situated, draws air down from the chamber through the space around the pipe in the floor. My cellar which was before damp, is now as dry and pleasant as any room in the house. Formerly articles placed in my cellar soon became mouldy, and were spoiled for want of ventilation.

"Another good way to accomplish the same object is to build the chimney into which the pipes from the stoves enter, from the cellar up with an opening in the callar. The heat rarifying the air causes an upward draught which effectually ventilates the cellar. We have tried this plan for years and know that it works well."

